# Long noncoding RNA ADAMTS9-AS1 represses ferroptosis of endometrial stromal cells by regulating the miR-6516-5p/GPX4 axis in endometriosis

**DOI:** 10.1038/s41598-022-04963-z

**Published:** 2022-02-16

**Authors:** Yiting Wan, Cancan Gu, Jueying Kong, Jin Sui, Ling Zuo, Yanhua Song, Jing Chen

**Affiliations:** Department of Gynecology, Shanghai Municipal Hospital of Traditional Chinese Medicine, Shanghai University of Traditional Chinese Medicine, No. 274 Middle Zhijiang Road, Shanghai, 200071 China

**Keywords:** Biochemistry, Cell biology, Genetics, Molecular biology

## Abstract

Endometriosis (EMs) is one of the most frequent diseases of reproductive-age women and is characterized by the growth of endometrial tissues beyond the uterus. The enhanced proliferative and migratory potential of endometrial stromal cells (ESCs) plays an important role in the progression of EMs. Mounting studies have demonstrated that long noncoding RNAs (lncRNAs) exert an important role in regulating the development and progression of EMs. Given the aberrant expression of lncRNA ADAMTS9-AS1 in ectopic endometrium (ecEM), we investigated the biological effect of ADAMTS9-AS1 on ESC proliferation and migration and explored the underlying mechanism. The current data showed that ADAMTS9-AS1 expression was significantly upregulated in ecEM compared with eutopic endometrium (euEM) in patients with EMs and in a murine model of EMs. Functionally, ADAMTS9-AS1 knockdown in ectopic ESCs (EESCs) decreased cell viability and migration, whereas ADAMTS9-AS1 overexpression in normal ESCs (NESCs) enhanced cell viability and migration. More importantly, the effect of ADAMTS9-AS1 inhibition on decreasing ESC viability was significantly blocked by ferrostatin-1 (Fer-1, a ferroptosis inhibitor), and ADAMTS9-AS1 overexpression repressed erastin (a ferroptosis activator)-induced cell death. Furthermore, the regulatory role of ADAMTS9-AS1 in ferroptosis was defined and evidenced by increased reactive oxygen species (ROS) levels and malonyl dialdehyde (MDA) content and decreased expression of glutathione peroxidase 4 (GPX4) after ADAMTS9-AS1 inhibition. Mechanistically, ADAMTS9-AS1 functioned as a competing endogenous RNA (ceRNA) by sponging miR-6516-5p to derepress the expression of GPX4, the critical repressor of ferroptosis. Taken together, these results demonstrate that upregulated ADAMTS9-AS1 accelerates ESC proliferation and migration by regulating miR-6516-5p/GPX4-dependent ferroptosis and may be a potential target for the treatment of EMs.

## Introduction

Endometriosis (EMs) is a common and polyfactorial disorder in which environmental, genetic, immunologic, and hormonal aberrations are correlated with the pathological process of EMs^1,2^. EMs is characterized by the growth of histologically normal endometrial tissues beyond the uterus and affects between 10 and 15% of women of fertile age worldwide^3,4^. The major clinical manifestations of EMs include infertility, chronic pelvic pain and vaginal bleeding and thus negatively affect the quality of life of these women. Recently, mounting experimental evidence has demonstrated that noncoding RNAs (ncRNAs) play an important regulatory role in the pathogenesis of EMs and in EMs-associated symptoms^5,6^.

Long noncoding RNA (lncRNA) is a kind of RNA molecule with a length of more than 200 nt that does not possess protein-coding potential^7^. An increasing number of studies have suggested that dysregulated lncRNA expression is closely correlated with a variety of biological processes and human diseases, such as chromosome dose compensation, development, tumorigenesis, and cardiovascular diseases^8–11^. For example, Xu et al. reported that 172 lncRNAs and 188 mRNAs are differentially expressed in endometrial carcinoma tissues compared with normal tissues^12^. Several lncRNAs (KIAA0087, RP11-501O2, FAM212B-AS1, etc.) might be a key regulator of endometrial carcinogenesis and progression. Emerging evidence has also revealed the role of lncRNAs in the pathogenesis of EMs. A total of 576 differentially expressed lncRNAs were identified in the ectopic endometrium (ecEM) of adenomyosis, of which 388 lncRNAs were increased and 188 lncRNAs were decreased^13^. Zhang et al. demonstrated that the expression of lncRNA CCDC144NL-AS1 is upregulated in ectopic endometrial tissues compared with paired eutopic endometrial and normal endometrial (NE) tissues^14^. Functional studies further showed that CCDC144NL-AS1 inhibition decreases endometrial stromal cell (ESC) migration and invasion^14^.

The role of lncRNA ADAMTS9-AS1 (hereafter named ADAMTS9-AS1) in tumor progression has been widely investigated. Low expression of ADAMTS9-AS1 predicts poorer survival than higher ADAMTS9-AS1 expression in patients with prostate cancer, and ADAMTS9-AS1 inhibits prostate cancer cell proliferation, indicating that ADAMTS9-AS1 functions as a tumor suppressor in prostate cancer^15^. A recent study showed that ADAMTS9-AS1 is significantly increased in ectopic endometrial tissues^16^. However, the biological role of ADAMTS9-AS1 in EMs remains unknown.

Ferroptosis is an iron-dependent nonapoptotic type of cell death that distinctly differs from other forms of cell death, including apoptosis, pyroptosis, senescence and autophagy^17–20^. Overproduction of iron-induced lipid reactive oxygen species (ROS) is the crucial event in ferroptosis^21,22^. Although the levels of iron and lipid peroxide content are increased in the peritoneal fluid of women with EMs compared to those without^23–25^, the role of ferroptosis in EMs has not been systematically investigated. In this study, we demonstrated that ADAMTS9-AS1 was significantly upregulated in ecEM compared with eutopic endometrium (euEM). ADAMTS9-AS1 knockdown decreased cell viability and migration, whereas the effect was blocked by a ferroptosis inhibitor. ADAMTS9-AS1 functioned as a competing endogenous RNA (ceRNA) by sponging miR-6516-5p to derepress the expression of GPX4.

## Materials and methods

### A murine model of EMs

Animal experiments were approved by the Experimental Animal Committee of Shanghai Municipal Hospital of Traditional Chinese Medicine and carried out according to Shanghai Municipal Hospital of Traditional Chinese Medicine guidelines for the use of animals. All animal experiments were performed in accordance with the ARRIVE guidelines as described previously^26^ to minimize the number of animals used in the study and animal suffering. Female BALB/c mice (4–6 weeks old, 18–20 g) were obtained from Shanghai Regan Biotechnology Co., Ltd. (Shanghai, China) and were reared in a specific, pathogen-free facility^27–29^. After 1 week of acclimatization, mice were randomly divided into two groups: the donor group (n = 10) and recipient groups (n = 10). Ovariosteresis and estradiol valerate injection (0.5 µg/mouse/week; Aladdin, Shanghai, China) was carried out to avoid differences in the estrous cycle^28^. Mice were anesthetized by 2% isoflurane, and then the ovaries on both sides were exposed through flank incisions and removed. Donor mice were sacrificed under isoflurane anesthesia, and each uterine horn of the donor mice was concentrated and peeled in warm PBS to remove uterine muscle. Endometrial tissues were weighed and cut into small fragments with scissors and resuspended in sterile PBS with 1 × ampicillin (Beyotime, Shanghai, China). After that, endometrium preparation was intraperitoneally injected into two recipient mice (50 mg/mouse). Two weeks after EM transplantation, endometriosis lesions and eutopic endometrial tissues were removed from the peritoneal cavities and uteri.

### Clinical samples

Human ectopic and eutopic endometrial tissues (n = 17) were provided by Shanghai Municipal Hospital of Traditional Chinese Medicine with written informed consent signed by the patients according to the Declaration of Helsinki Principles. All of the protocols in this study were approved by Shanghai Municipal Hospital of Traditional Chinese Medicine.

### Primary ESCs

Primary murine ESCs were isolated from the eutopic and ectopic endometrial tissues. Briefly, after digesting tissues with dispase to remove epithelial sheet^30^, endometrial tissues were washed with PBS, minced into small fragments, treated with 0.3% collagenase I and 0.01% trypsin (Sigma-Aldrich, MO, USA) in DMEM/12F medium (Sigma-Aldrich) for 50 min at 37 °C, and filtered through 100 μm and 70 μm nylon cell strainers. All the cells were maintained in DMEM/F-12 with 10% fetal bovine serum (Thermo Fisher Scientific, CA, USA) and 1 × penicillin–streptomycin (Beyotime) at 37 °C under humidified 5% CO_2_.

### Total RNA extraction and quantitative RT-PCR (qPCR) assay

Total RNA was extracted from endometrial tissues or ESCs with RNAiso Plus (TaKaRa, Shiga, Japan) and quantitated by Multiskan SkyHigh (Thermo Fisher Scientific). A Prime Script RT-PCR Kit (TaKaRa) was applied to carry out reverse transcription PCR (RT-PCR). The RT-PCR protocol was 65 °C for 5 min, 30 °C for 10 min, and 42 °C for 30 min. qPCR was performed using Accurate 96 (DLAB, China) with ChamQ Universal SYBR qPCR Master Mix (Vazyme, Nanjing, China). The program setting was based on the manufacturer’s recommendations, and the thermocycling conditions were as follows: initial denaturation at 95 °C for 10 s, denaturation at 95 °C for 10 s, and annealing and extension at 65 °C for 10 s; the reaction was performed for 30 cycles. Each reaction was carried out at least in triplicate. ADAMTS9-AS1 and GPX4 RNA transcription was quantified by the 2^−ΔΔCt^ method and normalized to endogenous β-actin mRNA. The primer sequences are listed in Supporting Table S1.

### Overexpression and RNA interference (RNAi)

The recombinant construct (pcDNA-ADAMTS9-AS1) containing the full-length ADAMTS9-AS1 sequence was constructed by inserting the open reading frame into the pcDNA vector. miR-6516-5p mimics were used to mimic the overexpression of miR-6516-5p. siRNA-ADAMTS9-AS1 (siADAMTS9-AS1) was used to knockdown ADAMTS9-AS1 in ESCs, and siRNA-control (N.C) was used as a control. The ESCs were seeded into culture dishes before transfection until the cells adhered on the second day. Lipofectamine 3000 (Invitrogen, Carlsbad, CA, USA) was applied to prepare a mixture of siADAMTS9-AS1, N.C, miR-6516-5p or pcDNA-ADAMTS9-AS1 with Lipofectamine 3000 according to the instructions.

### Cell viability assay

The viability of normal ESCs (NESCs) and ectopic ESCs (EESCs) was assessed with Cell Counting Kit (CCK)-8 (Solarbio, Beijing, China). Before transfection or drug treatment, ESCs were seeded onto 96-well plates at a density of 6 × 10^3^ cells/well and incubated for 24 h. To explore the effect of ADAMTS9-AS1 on regulating ESC viability and the pattern of ADAMTS9-AS1-induced ESC death, ADAMTS9-AS1 in ESCs was knocked down or overexpressed in the presence or absence of cell death inhibitors (Fer-1, 1 μM; ZVAD-FMK, 10 μM; necrostatin-1, 10 μM; 3-MA, 10 mM; and disulfiram, 5 μM) or inducers (erastin, 10 μM; sorafenib, 5 μM) purchased from Sigma-Aldrich. Then, the cells were treated with 15 µL CCK-8 reagent and incubated for another 3 h at 37 °C. The absorbance was measured at 450 nm with Multiskan SkyHigh (Thermo Fisher Scientific).

### Transwell migration assay

To explore the effect of ADAMTS9 AS1 and miR-6516-5p on regulating ESC migration, 24-well Transwell chambers (Corning, NY, USA) with 8-μm pores were used to assess the migration of EESCs. In brief, the transfected EESCs and NESCs were seeded into the upper chamber at a density of 200 μL 2.5 × 10^4^ cells with DMEM/12F medium without serum, and 700 μL of DMEM/12F medium supplemented with 10% FBS was added into the lower chamber. After incubating for 48 h at 37 °C with 5% CO_2_, the cells on the lower surface of the membranes were fixed with 4% paraformaldehyde (Beyotime) and stained with 0.1% crystal violet (Beyotime) for 10 min. Images were obtained using BZ-X700 (Keyenth, Osaka, Japan), and the number of cells migrating to independent areas was counted using Fiji software (ImageJ 1.51s, NIH, USA).

### TUNEL staining

Cell death was assessed using terminal deoxynucleotidyl transferase dUTP nick end labelling (TUNEL). In brief, after being transfected with si-ADAMTS9-AS1 or siRNA-control for 48 h, EESCs were fixed in 4% formaldehyde and then stained using the TUNEL mixture (Elabscience, Wuhan, China) for 60 min at 37 °C. Cell nuclei was counterstained with DAPI (Beyotime) for 5 min. Cells were observed using a fluorescence microscopy (DMI4000B, Leica).

### Measure of iron concentration

Intracellular iron concentrations were measured using an iron assay kit (ab83366, Abcam) according to the manufacturer’s instructions. Briefly, ESCs were lysed with iron assay buffer (3 × 10^3^ cells/well) in a final volume of 100 μL, treated with 5 μL of iron reducer, plated in 96-well plates and incubated at room temperature for 30 min. Then, 100 μL of iron probe was added to mark ferrous ions. The absorbance was measured at 593 nm with a Multiskan SkyHigh (Thermo Fisher Scientific).

### GSH and MDA content

The malondialdehyde (MDA) content of the ESCs was assayed using a lipid peroxidation assay kit (ab118970, Abcam, MA, USA) after ADAMTS9-AS1 knockdown or overexpression in the presence or absence of miR-6516-5p or siGpx4. The glutathione (GSH) content in the ESCs was assayed using a glutathione assay kit (ab65322, Abcam).

### Lipid ROS level

Lipid ROS levels were tested using a fluorescent-labeled oxidation-sensitive probe (C11-BODIPY, Thermo Fisher Scientific) as previously described^31^. In brief, ESCs (5 × 10^5^) were plated in 24-well plates and treated with the indicated reagents. After 48 h, the ESCs were treated with 1.5 μM C11-BODIPY for 30 min at 37 °C. Lipid ROS levels were assessed using a flow cytometer (CytoFLEX, Beckman Coulter, FL, USA).

### Western blotting

The protein extracted from the ESC lysates was quantified using the BCA Protein Assay Kit (Beyotime). Equal amounts of protein (approximately 50 μg) were loaded and separated by 12% SDS-PAGE for each experiment and then transferred to PVDF membranes (0.45 μm pore size; Millipore Corp., MA, USA). After blocking in 3% BSA at room temperature for 1 h, the membranes were treated with primary antibodies against GPX4 (1:4000, ab125066, Abcam) and β-actin (1:5000, ab8226, Abcam) for 18 h at 4 °C, followed by incubation with HRP-conjugated secondary antibody (HRP-labeled goat anti-rabbit IgG (H + L); 1:5000; Beyotime) for 1.5 h. The bands were visualized using an ECL reagent (Beyotime) and quantified using Fiji software.

### Fluorescence in situ hybridization (FISH)

The subcellular localization of ADAMTS9-AS1 was assessed with a FISH Tag RNA Multicolor Kit (Carlsbad, CA, USA). ESCs (3 × 10^4^/well) were loaded on cover glasses and fixed with 4% paraformaldehyde (pH = 7.4) for 5 min. After fixation, the ESCs were digested with protease K (3 μg/mL) with glycine and acetic anhydride, dehydrated in 75, 95, and 100% ethanol for 3 min each, air-dried at room temperature, and hybridized at 37 °C with probes (150 μL, 450 ng/mL) against ADAMTS9-AS1 for 48 h. Finally, the ESCs were stained with DAPI (Thermo Fisher Scientific) and observed under a fluorescence scanning microscope (BZ-X700; Keyenth, Osaka, Japan).

### Luciferase assay

The recombinant plasmids of pGL3-ADAMTS9-AS1 and pGL3-GPX4-3’UTR or their mutants were constructed in our laboratory by cloning approximately 350 bp of cDNA into the pGL3 vector (Vector Builder, Guangzhou, China). The ESCs (4.5 × 10^4^) were plated into 48-well plates and cotransfected with 70 nM miR-6516-5p or miR-cont (used as a control) with Lipofectamine 3000. Luciferase activity was detected using the Renilla-Lumi Luciferase Reporter Gene Assay Kit (Beyotime, Shanghai, China) according to the manufacturer’s protocol.

### RNA-pulldown assay

Biotin-labeled miR-6516-5p and miR-cont were transcribed in vitro using the Pierce Magnetic RNA–Protein Pull-Down Kit (Thermo Fisher Scientific). Approximately 2 × 10^7^ cells were dissolved in standard lysis buffer (Thermo Fisher Scientific) plus 10 U/mL RNase inhibitor (Beyotime). Next, streptavidin-labeled beads were added to each binding reaction and incubated for 90 min. Finally, ADAMTS9 AS1 in the eluate was quantified by qPCR.

### Statistical analysis

The data were analyzed using GraphPad Prism v.7.0 (CA, USA) and presented as the mean ± SEM from at least three independent experiments. The significance between the different groups was assessed using Student’s *t* test or one-way ANOVA followed by a Tukey–Kramer multiple comparisons test. P values less than 0.05 were considered to indicate a statistically significant difference.

## Results

### ADAMTS9-AS1 expression was significantly upregulated in ecEM

**Figure 1:**
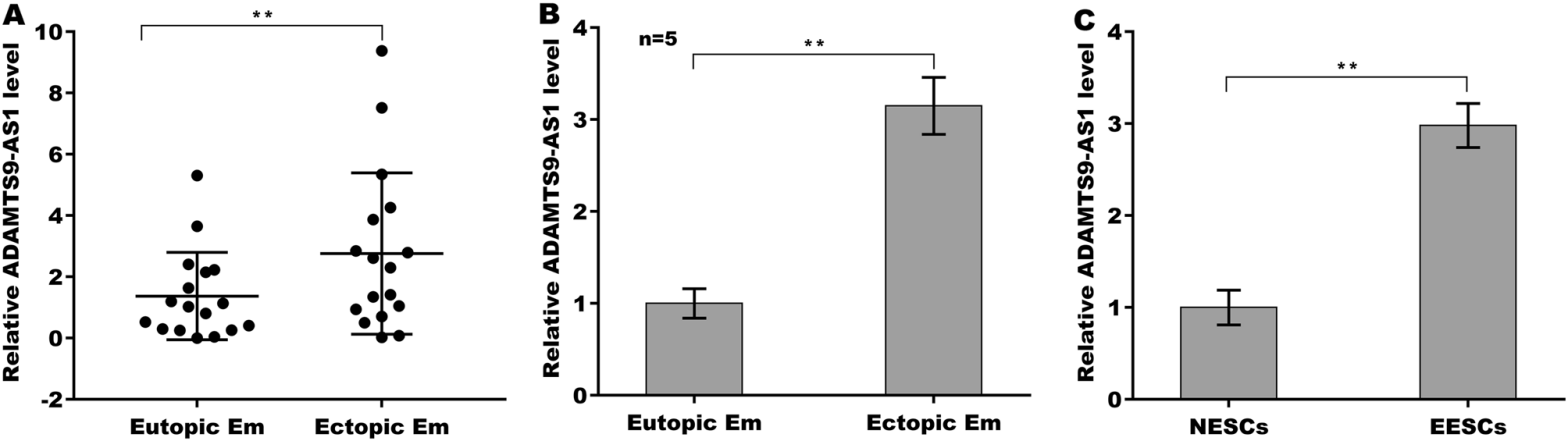
ADAMTS9-AS1 expression was increased in ecEM tissues. (**A**) qPCR analysis of ADAMTS9-AS1 in paired ecEM and euEM tissues (n = 17) in women with EMs. (**B**) A murine model of EMs was established, and the expression level of ADAMTS9-AS1 was assessed using qPCR analysis in ecEM and euEM tissues (n = 5). (**C**) Primary EESCs and NESCs were isolated from ecEM and euEM tissues of EMs mice, respectively, and then the ADAMTS9-AS1 level was assessed using qPCR analysis (n = 5). **p < 0.01.

The expression level of ADAMTS9-AS1 is dysregulated in ecEM^16^ and in several types of cancer^15,32,33^. To investigate the biological function of ADAMTS9-AS1 in EMs, the expression pattern of ADAMTS9-AS1 was verified in endometrial tissues. The data from qPCR analysis in seventeen paired ecEM and euEM tissues showed that the expression level of ADAMTS9-AS1 was significantly increased in ecEM compared to euEM (Fig. 1A). To further validate the results, a murine model of EMs was established, and ecEM and euEM tissues were collected. Figure 1B showed that ADAMTS9-AS1 expression was also upregulated in ecEM compared to euEM in a murine model of EMs. Then, primary EESCs and NESCs were collected from the ecEM and euEM tissues, respectively, and the ADAMTS9-AS1 level was assessed. As shown in Fig. 1C, the ADAMTS9-AS1 level in the EESCs was higher than that in the NESCs.

### ADAMTS9-AS1 accelerated ESC growth and migration

**Figure 2:**
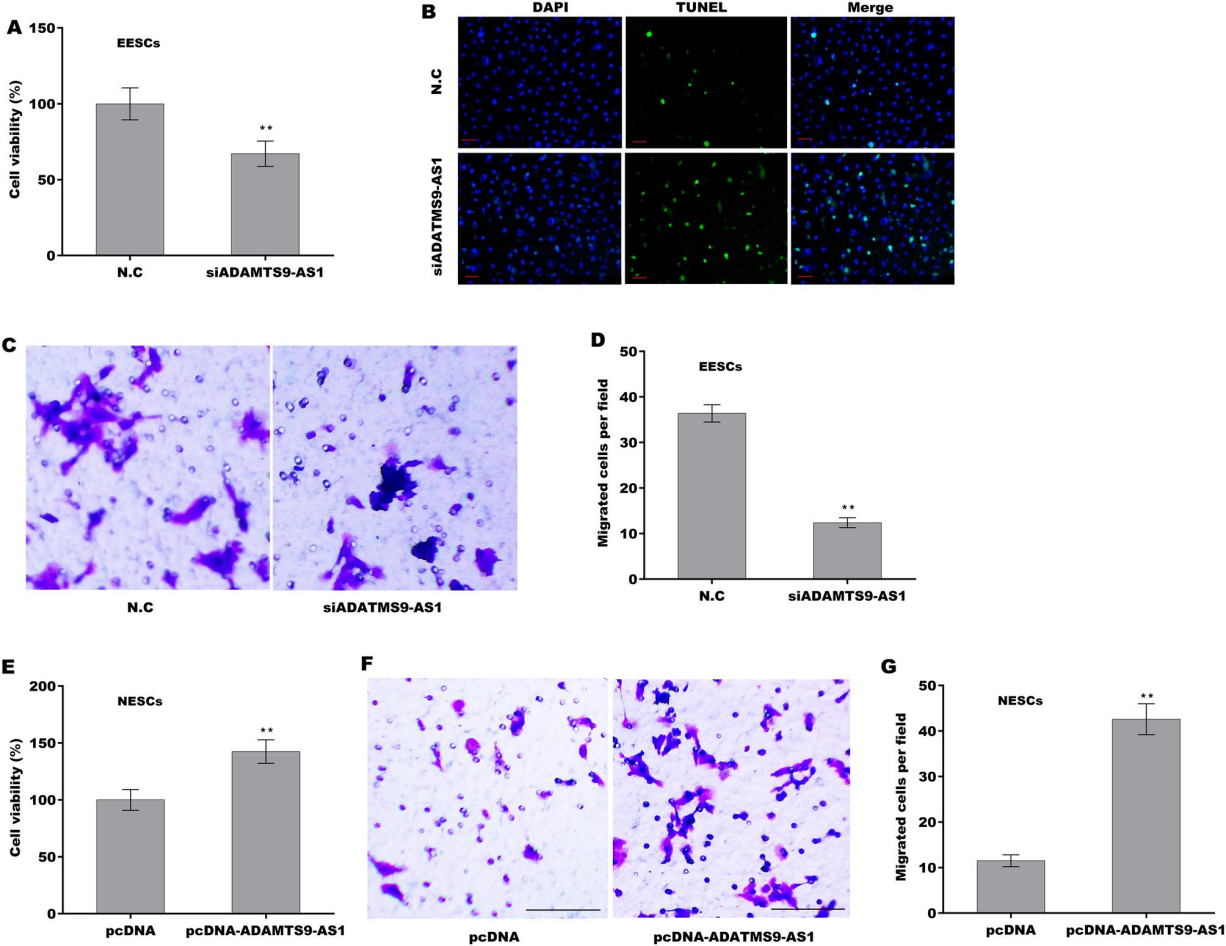
ADAMTS9-AS1 facilitated ESC growth and migration. (**A**) Cell viability of EESCs after ADAMTS9-AS1 knockdown was assessed using a CCK-8 assay. (**B**) Cell death of EESCs after ADAMTS9-AS1 knockdown was assessed using Tunel assay. Scan bar = 50 µM. (**C**,**D**) Cell migration of EESCs after ADAMTS9-AS1 knockdown was assessed using a Transwell migration assay. (**E**) Cell viability of NESCs after ADAMTS9-AS1 overexpression was assessed using a CCK-8 assay. (**F**,**G**) Cell migration of NESCs after ADAMTS9-AS1 overexpression was assessed using a Transwell migration assay. **p < 0.01.

To explore the effect of ADAMTS9-AS1 on regulating ESC viability and migration, ADAMTS9-AS1 was knocked down in EESCs and overexpressed in NESCs (Supporting Fig. S1A,B). Figure 2A showed that ADAMTS9-AS1 knockdown in EESCs significantly decreased cell viability. The results from Tunel assay revealed that ADAMTS9-AS1 knockdown resulted in obvious cell death (Fig. 2B). In addition, the migratory behavior of ESCs plays a crucial role in EMs pathogenesis^34^. Figure 2C,D showed that ADAMTS9-AS1 knockdown in EESCs significantly repressed cell migration. In contrast, ADAMTS9-AS1 overexpression in NESCs increased cell viability (Fig. 2E) and facilitated cell migration (Fig. 2F,G). These data suggest that upregulated ADAMTS9-AS1 increases ESC viability and promotes ESC migration.

### ADAMTS9-AS1 repressed ferroptosis of ESCs

**Figure 3:**
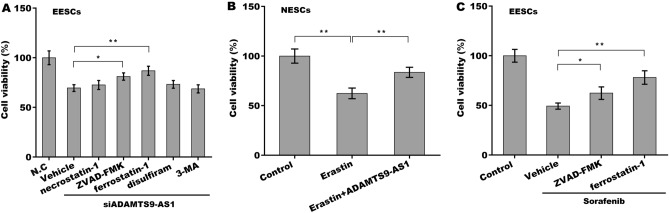
ADAMTS9-AS1 increased ESC viability by regulating ferroptosis. (**A**) Cell viability of EESCs was assessed after ADAMTS9-AS1 knockdown using a CCK-8 assay in the presence of the indicated inhibitors (ZVAD-FMK, 10 μM; necrostatin-1, 10 μM; ferrostatin-1, 1 μM; disulfiram, 5 μM; 3-MA, 10 mM). (**B**) Cell viability of NESCs was assessed after ADAMTS9-AS1 overexpression using a CCK-8 assay in the presence or absence of erastin (10 μM). (**C**) Cell viability of EESCs was assessed after treatment with the indicated inhibitors (ZVAD-FMK, 10 μM; ferrostatin-1, 1 μM) in the presence or absence of sorafenib (5 μM). *p* < 0.05, **p < 0.01.

To investigate the pattern of ADAMTS9-AS1-induced ESC death, EESCs were treated with ADAMTS9-AS1-specific siRNA (siADAMTS9-AS1) in the presence of Fer-1 (a specific inhibitor of ferroptosis), ZVAD-FMK (a specific inhibitor of apoptosis), 3-MA (a specific inhibitor of autophagy), or disulfiram (a specific inhibitor of pyroptosis). Forty-eight hours later, cell viability was assessed using CCK-8. Figure 3A showed that the siADAMTS9-AS1-mediated decrease in EESC viability was significantly prevented by Fer-1 and ZVAD-FMK but not necrostatin-1, 3-MA or disulfiram, suggesting that ADAMTS9-AS1 knockdown accelerated EESC death by triggering apoptosis and ferroptosis. The role of ADAMTS9-AS1 in regulating ferroptosis of EESCs was next explored because the effect of Fer-1 on regulating EESC viability was more significant than that of ZVAD-FMK. Figure 3B further revealed that erastin (a specific activator of ferroptosis) treatment decreased NESC viability, whereas ADAMTS9-AS1 overexpression blocked this effect. Fer-1 also prevented sorafenib-induced EESC death (Fig. 3C). These data demonstrate that ferroptosis is an important form of ESC death and that ADAMTS9-AS1 is negatively correlated with ESC ferroptosis.

**Figure 4:**
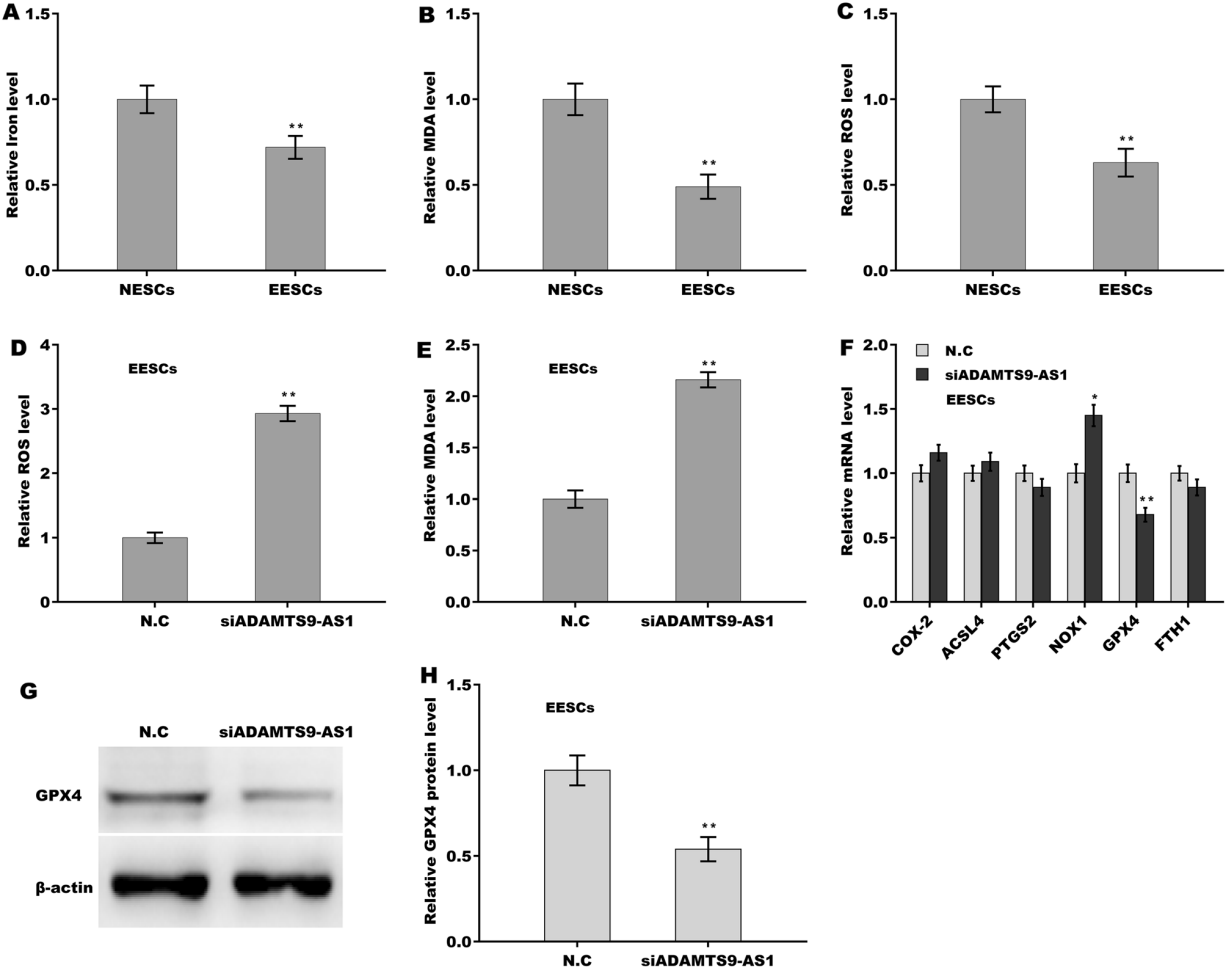
ADAMTS9-AS1 repressed ferroptosis of ESCs by regulating GPX4. The Fe^2+^ levels (**A**), MDA content (**B**), and ROS levels (**C**) were assayed using the indicated kits for NESCs and EESCs. The ROS levels (**D**) and MDA content (**E**) were assessed using the indicated kits for EESCs after ADAMTS9-AS1 knockdown. (**F**) The mRNA levels of COX-2, ACSL4, PTGS2, NOX1, GPX4, and FTH1 were assayed using qPCR analysis in EESCs after ADAMTS9-AS1 knockdown. (**G**,**H**) The protein level of GPX4 was assayed using western blot analysis in EESCs after ADAMTS9-AS1 knockdown. *p < 0.05, **p < 0.01.

We then compared the ferroptosis level between NESCs and EESCs. Figure 4A–C showed that the relative iron concentration, MDA content, and ROS level were lower in EESCs than in NESCs, indicating that ferroptosis was repressed in EESCs. To determine whether ADAMTS9-AS1 knockdown triggered ferroptosis of EESCs, ROS levels and MDA content were assessed after ADAMTS9-AS1 knockdown. Figure 4D,E showed that ADAMTS9-AS1 inhibition resulted in a significant increase in ROS and MDA levels, suggesting that ADAMTS9-AS1 negatively regulated ferroptosis of EESCs. To uncover the mechanism by which ADAMTS9-AS1 repressed ferroptosis, the expression level of ferroptosis-related genes (COX-2, ACSL4, PTGS2, NOX1, GPX4, and FTH1) were assayed in EESCs after ADAMTS9-AS1 depletion. Figure 4F–H showed that ADAMTS9-AS1 depletion significantly decreased the mRNA and protein expression levels of GPX4, a key repressor of ferroptosis. These data indicate that ADAMTS9-AS1 negatively regulates ferroptosis of ESCs, possibly by regulating GPX4.

### ADAMTS9-AS1 acted as a ceRNA by sponging miR-6516-5p

**Figure 5:**
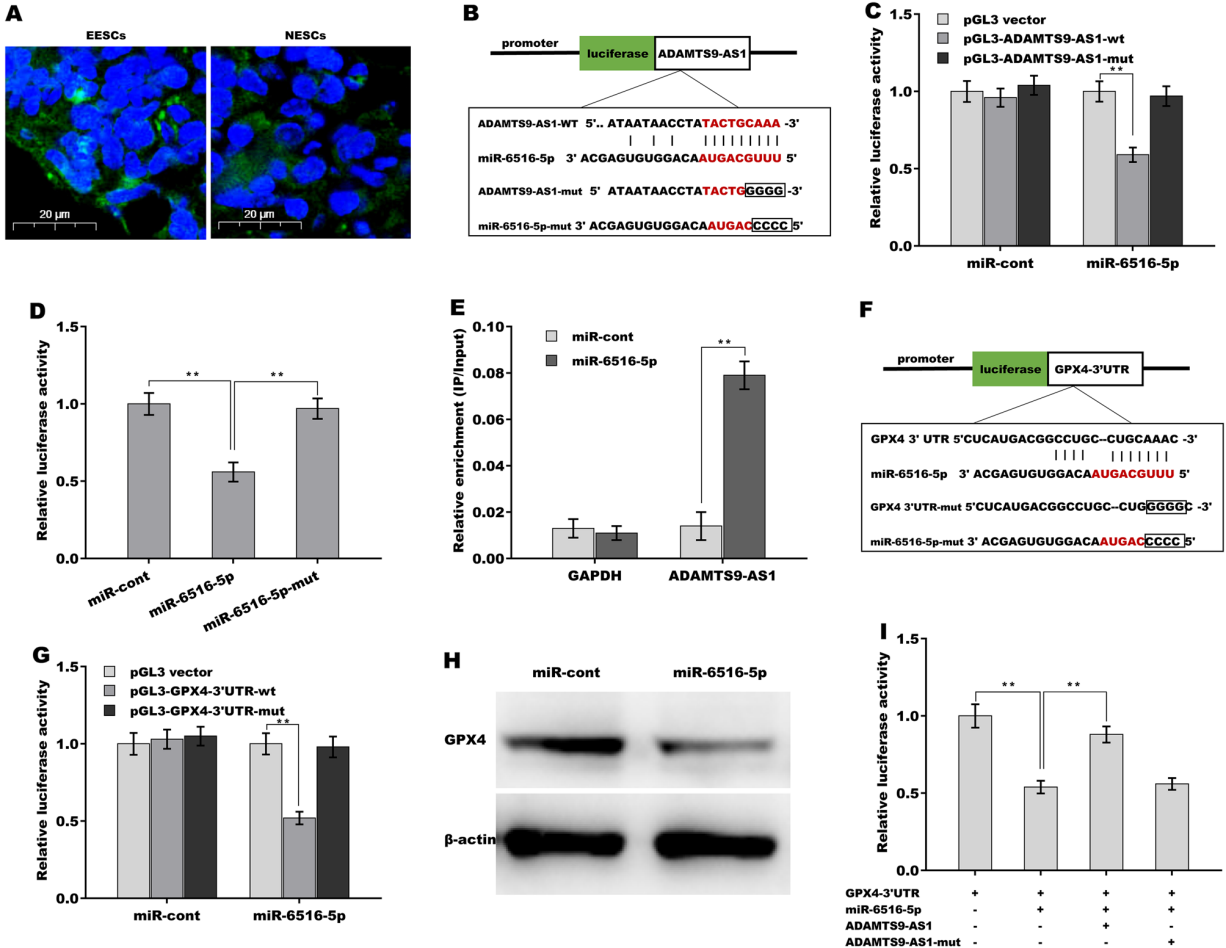
ADAMTS9-AS1 acted as a ceRNA by sponging miR-6516-5p. (**A**) The subcellular localization of ADAMTS9-AS1 in EESCs and NESCs was analyzed using an RNA-FISH assay. Probes targeting ADAMTS9-AS1 were stained green, and DAPI was used to stain the nuclei of EESCs and NESCs. (**B**) Schematic representation of the predicted binding sites of miR-6516-5p in the ADAMTS9-AS1 sequence. (**C**) Luciferase activity was assessed using a dual-luciferase reporter assay in ESCs cotransfected with miR-6516-5p and recombinant luciferase reporter plasmids containing ADAMTS9-AS-wt (or ADAMTS9-AS-mut). Renilla luciferase was used as the internal control for normalizing transfection efficiency, and the data are shown as the relative ratio of firefly luciferase activity. (**D**) Luciferase activity was assessed using a dual-luciferase reporter assay in ESCs cotransfected with miR-6516-5p (or miR-6516-5p-mut or miRcont) and recombinant luciferase reporter plasmids containing ADAMTS9-AS-wt. (**E**) The direct combination of ADAMTS9-AS with miR-6516-5p was assessed through an RNA pull-down assay using 3′-biotinylated miR-6516-5p as bait. (**F**) Schematic representation of the predicted binding sites of miR-6516-5p in the GPX4-3’UTR sequence. (**G**) Luciferase activity was assessed using a dual-luciferase reporter assay in ESCs cotransfected with miR-6516-5p (or miRcont) and recombinant luciferase reporter plasmids containing GPX4-3’UTR-wt (or GPX4-3’UTR-mut). (**H**) The protein expression of GPX4 was assessed using western blot analysis in ESCs after miR-6516-5p overexpression. (**I**) Luciferase activity was assessed using a dual-luciferase reporter assay in ESCs cotransfected with miR-6516-5p and luciferase reporters containing GPX4-3’UTR, ADAMTS9-AS-wt, or ADAMTS9-AS-Mut. *p < 0.05, **p < 0.01.

LncRNAs located in the cytoplasm commonly function as ceRNAs to indirectly control mRNA expression by absorbing miRNAs^35,36^. To explore the mechanism underlying ADAMTS9-AS1 in regulating ferroptosis, the subcellular localization of ADAMTS9-AS1 in ESCs was first assessed using FISH. As shown in Fig. 5A, ADAMTS9-AS1 was primarily located in the cytoplasm in EESCs and NESCs. The bioinformatics tool miRDB (http://mirdb.org/cgi-bin/search.cgi)^37^ was used to predict the potential miRNAs absorbed by ADAMTS9-AS1. A total of 104 miRNAs were predicted through the miRDB tool (Supporting Table S2). Based on the above results that ADAMTS9-AS1 positively regulated GPX4 expression, miRNAs targeting GPX4 were screened using TargetScan 7.2 (http://www.targetscan.org/vert_72/). miR-6516-5p exhibited the potential to be involved in ADAMTS9-AS1/miR-6516-5p/GPX4 ceRNA networks (Fig. 5B,F).

To assess the direct combination of ADAMTS9-AS1 with miR-6516-5p, the recombinant plasmids of pGL3-ADAMTS9-AS1 or pGL3-ADAMTS9-AS1-mut were transiently transfected into ESCs in the presence of miR-6516-5p. Figure 5C revealed that the luciferase activity of pGL3-ADAMTS9-AS1 was obviously reduced after cotransfection with miR-6516-5p compared with the control (miR-cont), whereas miR-6516-5p did not decrease the luciferase activity of pGL3-ADAMTS9-AS1-mut. Similarly, mutant miR-6516-5p did not affect the luciferase activity of pGL3-ADAMTS9-AS1 (Fig. 5D), indicating a specific combination of miR-6516-5p with ADAMTS9-AS1 by base-pair complementarity in the seed region of miR-6516-5p. An RNA pull-down assay was used to further verify the direct binding of miR-6516-5p with ADAMTS9-AS1. Figure 5E revealed that ADAMTS9-AS1 was more enriched in the miR-6516-5p immunoprecipitates than in the miR-cont immunoprecipitates.

Based on the prediction that miR-6516-5p potentially targets GPX4 (Fig. 5F), we next determined whether miR-6516-5p regulates GPX4 expression at the posttranscriptional level. To this end, the recombinant plasmids of pGL3-GPX4-3’UTR or pGL3-GPX4-3’UTR-mut were transfected into ESCs in the presence of miR-6516-5p. Figure 5G revealed that the luciferase activity of pGL3-GPX4-3’UTR was obviously reduced after cotransfection with miR-6516-5p compared with the control (miR-cont), whereas miR-6516-5p did not decrease the luciferase activity of pGL3-GPX4-3’UTR-AS1-mut. Although miR-6516-5p overexpression (Supporting Fig. S1C) could not inhibit GPX4 mRNA expression (data not shown), miR-6516-5p significantly repressed the protein expression of GPX4 (Fig. 5H). Then, the regulatory correlation of ADAMTS9-AS1 with miR-6516-5p and GPX4 was explored. As shown in Fig. 5I, the luciferase activity of pGL3-GPX4-3’UTR was decreased following miR-6516-5p overexpression, but the suppressive effect was destroyed in the presence of ADAMTS9-AS1, not ADAMTS9-AS1-Mut. These results demonstrate that ADAMTS9-AS1 competitively binds to miR-6516-5p with GPX4 and that ADAMTS9-AS1 might act as a ceRNA to regulate GPX4 expression by sponging miR-6516-5p.

### ADAMTS9-AS1 increased GPX4 expression in a miR-6516-5p-dependent manner

**Figure 6:**
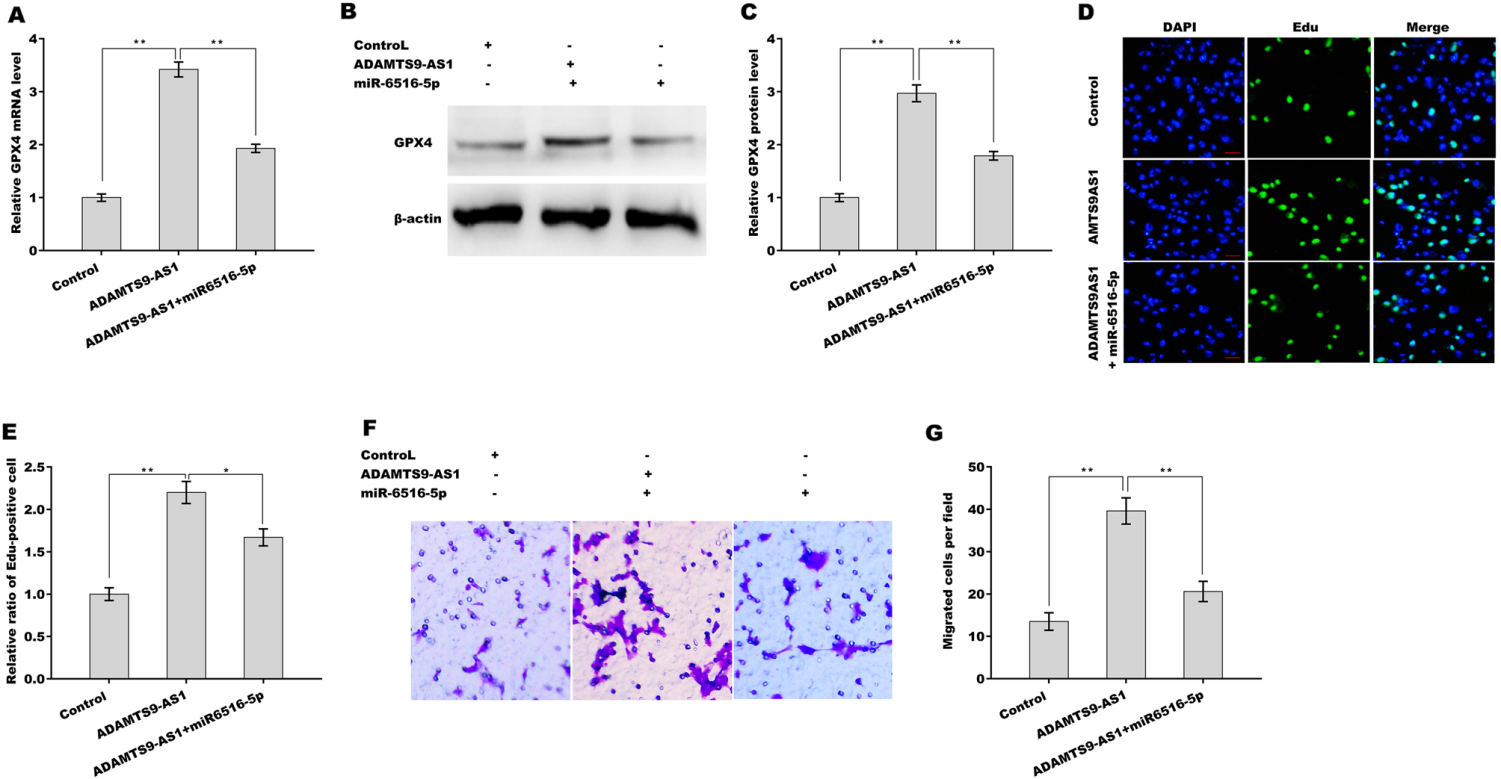
ADAMTS9-AS1 increased GPX4 expression in a miR-6516-5p-dependent manner. qPCR (**A**) and western blot analysis (**B**,**C**) of GPX4 expression in ESCs after overexpression of ADAMTS9-AS1 in the presence or absence of miR-6516-5p. (**D**,**E**) ESC proliferation was assessed using an Edu assay after overexpression of ADAMTS9-AS1 in the presence or absence of miR-6516-5p. Scan bar = 50 µM. (**F**,**G**) ESC migration was assessed using a Transwell migration assay after overexpression of ADAMTS9-AS1 in the presence or absence of miR-6516-5p. *p < 0.05. **p < 0.01.

**Figure 7:**
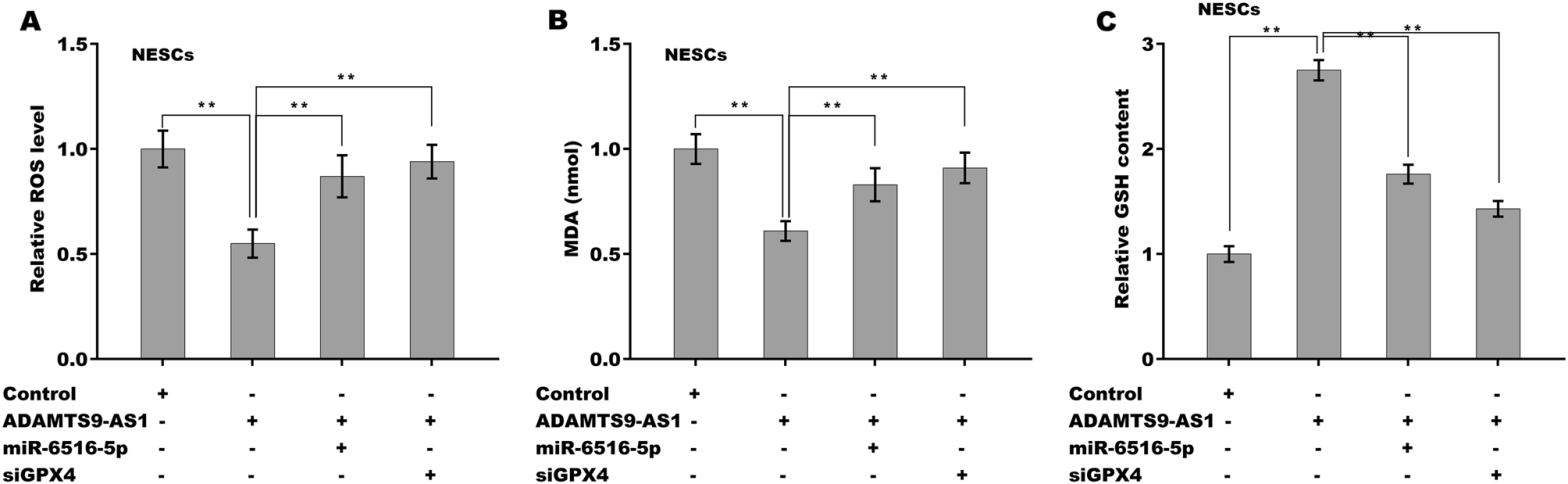
The effect of the ADAMTS9-AS1/miR-6516-5p/GPX4 axis on ferroptosis. The ROS levels (**A**), MDA content (**B**), and GSH content (**C**) were assessed in NESCs after ADAMTS9-AS1 overexpression in the presence or absence of miR-6516-5p or siRNA-Gpx4. **p < 0.01.

To verify whether ADAMTS9-AS1 functioned as a ceRNA to increase GPX4 expression by sponging miR-6516-5p, ADAMTS9-AS1 was overexpressed in ESCs in the presence or absence of miR-6516-5p, and GPX4 expression was assessed using qPCR and western blot analysis. As shown in Fig. 6A–C, forced expression of ADAMTS9-AS1 increased the mRNA and protein levels of GPX4 in ESCs, whereas miR-6516-5p reversed this effect. Functionally, ADAMTS9-AS1 enhanced the proliferative (Fig. 6D,E) and migratory (Fig. 6F,G) ability of ESCs, whereas miR-6516-5p overexpression significantly inhibited this effect (Fig. 6D–G). More importantly, ADAMTS9-AS1 decreased ROS levels and MDA content and increased GSH content, whereas miR-6516-5p overexpression or GPX4 knockdown significantly blocked this effect (Fig. 7A–C), indicating that ADAMTS9-AS1 repressed ferroptosis in a miR-6516-5p/GPX4-dependent manner. Taken together, the present data demonstrate that ADAMTS9-AS1 accelerates EMs progression by repressing miR-6516-5p/GPX4-dependent ferroptosis of ESCs.

## Discussion

Iron (Fe) is an indispensable element that regulates cell survival and proliferation, and it has been reported that Fe shortages are correlated with many reproductive diseases^20^. Paradoxically, Fe overload exerts multiple effects on EMs progression, including oxidative stress or lesion proliferation^38^. As an Fe-dependent nonapoptotic form of cell death, the biological role of ferroptosis in EMs is gradually being revealed. Ferroptosis resistance is correlated with ESC growth and proliferation, and ferroptosis-associated genes may possess clinical potential in the diagnosis and treatment of EMs. In the present study, we demonstrate that (i) ADAMTS9-AS1 expression was significantly increased in the ecEM, (ii) ADAMTS9-AS1 accelerated ESC growth and migration, (iii) ADAMTS9-AS1 repressed ferroptosis of ESCs, (iv) ADAMTS9-AS1 acted as a ceRNA by sponging miR-6516-5p, and (v) ADAMTS9-AS1 increased GPX4 expression in a miR-6516-5p-dependent manner. These results demonstrated the important function of the ADAMTS9-AS1/miR-6516-5p/GPX4/ferroptosis axis in regulating ESC viability and migration and may provide a potential opportunity for EMs therapy.

The role of ADAMTS9-AS1 in tumorigenesis and progression has been widely investigated. Through lncRNA expression profiling analysis, Wang et al. first demonstrated that the ADAMTS9-AS1 level is significantly increased in epithelial ovarian cancer tissues compared with normal tissues^39^. ADAMTS9-AS1 overexpression represses prostate cancer cell proliferation, and low expression levels of ADAMTS9-AS1 predict poorer survival than higher ADAMTS9-AS1 expression levels, suggesting that ADAMTS9-AS1 functions as a tumor suppressor in prostate cancer^15^. In contrast, ADAMTS9-AS1 facilitates cancer cell proliferation and migration in hepatocellular carcinoma and colorectal cancer^40,41^. These studies indicate that ADAMTS9-AS1 might exert different biological effects in different types of cells. In particular, a recent study showed that ADAMTS9-AS1 expression is dysregulated in ectopic endometrial tissues^16^. Based on these facts, we investigated the biological role of ADAMTS9-AS1 in the pathogenesis of EMs. The current results revealed that ADAMTS9-AS1 expression is significantly increased in ecEM compared with euEM in patients with EMs and in a murine model of EMs. Functional studies demonstrated that ADAMTS9-AS1 inhibition in ESCs represses cell viability and migration, whereas ADAMTS9-AS1 overexpression increases cell viability and migration, indicating that ADAMTS9-AS1 functions as an activator of EMs.

The underlying mechanism by which ADAMTS9-AS1 facilitates ESC viability was further investigated. As an antisense lncRNA, ADAMTS9-AS1 cannot affect the expression of overlapping coding genes (ADAMTS9, data not shown)^39^, indicating that ADAMTS9-AS1 possesses other modes of function in EMs. Emerging studies have demonstrated that lncRNAs can interact with miRNAs as ceRNAs to regulate target mRNAs^42^. The ceRNA hypothesis has also been confirmed in EMs. Wang et al. identified an EMs-related ceRNA network involving forty-five pathways and several ceRNAs as potential biomarkers for endometrial receptivity^43^. LINC01541 sponges miR-506-5p to regulate its downstream Wnt/β-Catenin pathway in EMs and thus inhibits the proliferation and invasion of ESCs^44^. In this study, we demonstrated that ADAMTS9-AS1 functions as a ceRNA by sponging miR-6516-5p to derepress the expression of GPX4.

It is well known that GPX4 is the critical repressor of ferroptosis^45^. As a glutathione-dependent enzyme, GPX4 converts toxic lipid hydroperoxides to nontoxic lipid alcohols^46^ and thus decreases Fe-induced conversion of lipid hydroperoxides to highly reactive lipid alkoxy radicals. The genetic variants of *GPX4* are different between women with advanced stage EMs and mild EMs^47^. For example, GPX4 rs713041 is associated with the severity of EMs, indicating that abnormal GPX4 is involved in the pathogenesis of EMs. Moreover, the regulatory role of lncRNAs in GPX4 expression has been verified. Bai et al. demonstrated that H19 knockdown decreases GPX4 expression and facilitates ferroptosis in spontaneous abortion^48^. Based on these analyses, we investigated whether ADAMTS9-AS1 regulates ferroptosis resistance in EMs through the miRNA/GPX4 axis. We demonstrated that ADAMTS9-AS1 accelerates ESC proliferation and migration by regulating miR-6516-5p/GPX4-dependent ferroptosis.

## Supplementary Information

Supplementary Information 1.

Supplementary Information 2.

Supplementary Information 3.

Supplementary Information 4.

## Data Availability

All data generated or analyzed during this study are included in this published article (and its Supplementary Information files).
