# Supplementary material for: Long noncoding RNA ADAMTS9-AS1 represses ferroptosis of endometrial stromal cells by regulating the miR-6516-5p/GPX4 axis in endometriosis
Source: Sci Rep. 2022 Feb 16;12:2618. doi: 10.1038/s41598-022-04963-z (PMC8850595; doi:10.1038/s41598-022-04963-z)
Supplement: Supplementary file 1 — Supplementary Information 1. [file 41598_2022_4963_MOESM1_ESM.pptx]

## Slide 1
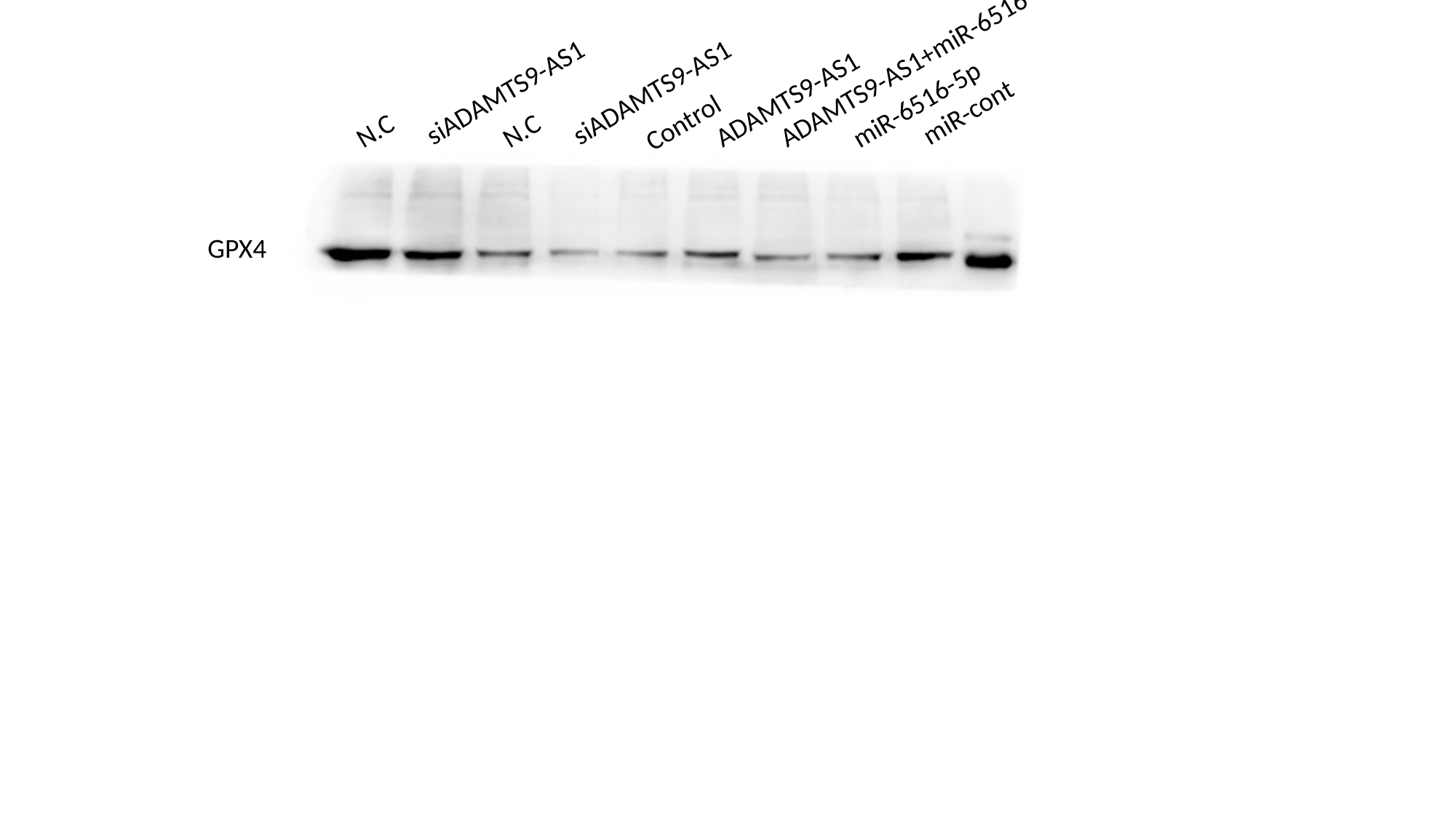

miR-6516-5p
 miR-cont
N.C
 siADAMTS9-AS1
N.C
 siADAMTS9-AS1
Control
 ADAMTS9-AS1
 ADAMTS9-AS1+miR-6516-5p
GPX4

## Slide 2
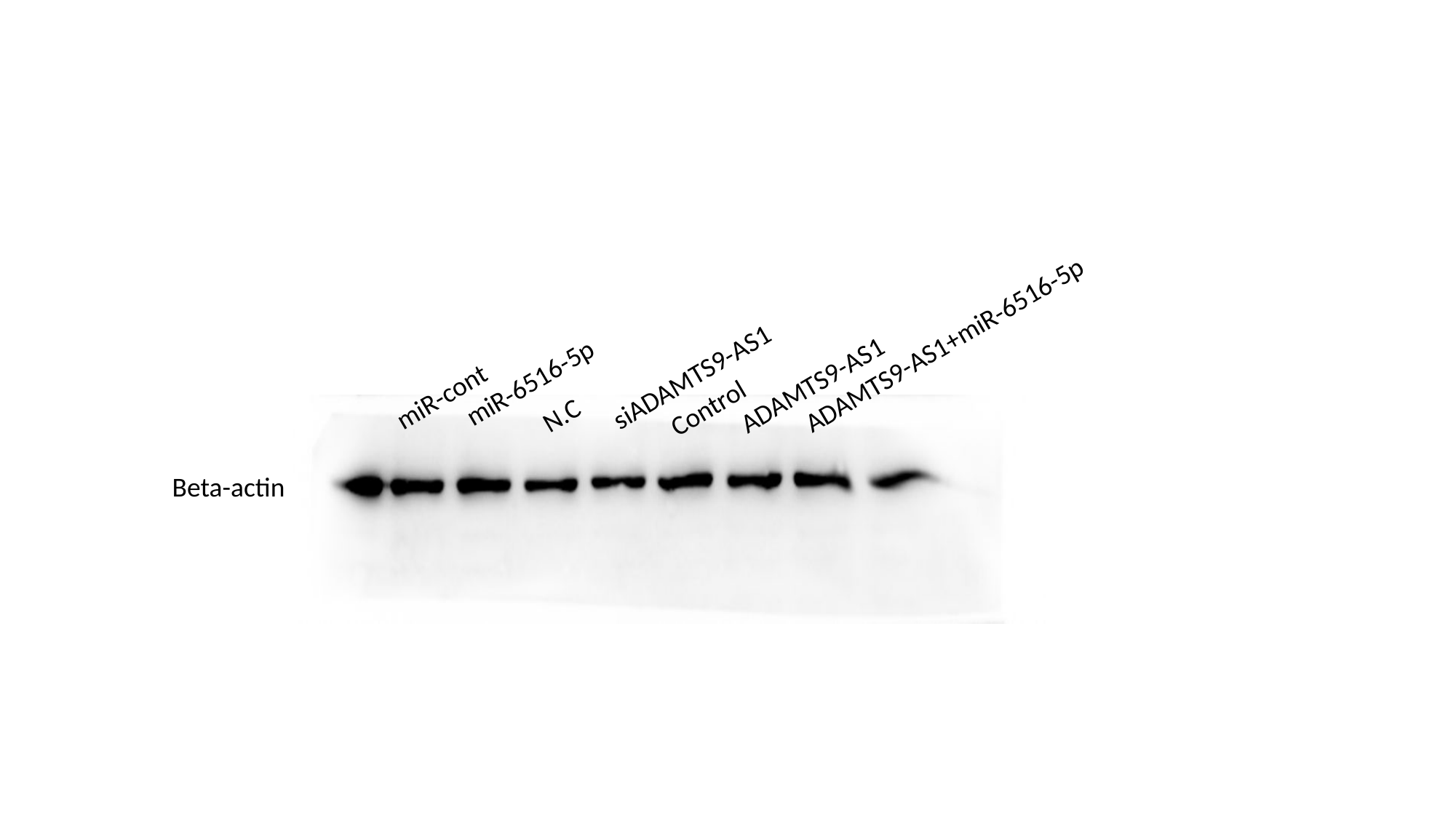

miR-cont
 miR-6516-5p
N.C
 siADAMTS9-AS1
Control
 ADAMTS9-AS1
 ADAMTS9-AS1+miR-6516-5p
Beta-actin
