# Supplementary figures and images for: Long noncoding RNA ADAMTS9-AS1 represses ferroptosis of endometrial stromal cells by regulating the miR-6516-5p/GPX4 axis in endometriosis
Source: Sci Rep. 2022 Feb 16;12:2618. doi: 10.1038/s41598-022-04963-z (PMC8850595; doi:10.1038/s41598-022-04963-z)

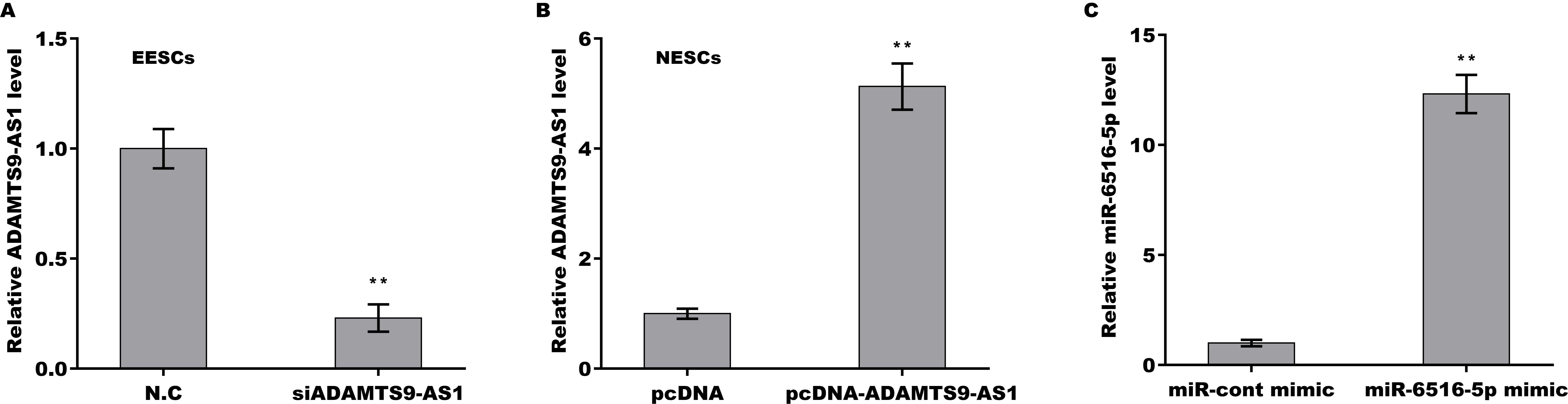

Supplement: Supplementary file 2 — Supplementary Information 2. [file 41598_2022_4963_MOESM2_ESM.tif]
