# Supplementary material for: Long noncoding RNA ADAMTS9-AS1 represses ferroptosis of endometrial stromal cells by regulating the miR-6516-5p/GPX4 axis in endometriosis
Source: Sci Rep. 2022 Feb 16;12:2618. doi: 10.1038/s41598-022-04963-z (PMC8850595; doi:10.1038/s41598-022-04963-z)
Supplement: Supplementary file 3 — Supplementary Information 3. [file 41598_2022_4963_MOESM3_ESM.docx]

**Supporting Table S1. Primer, siRNA, or miRNA sequence used in the study**

|  |  |  |
| --- | --- | --- |
|  | Sense (5’-3’) | Antisense (5’-3’) |
| pcDNA-ADAMTS9-AS1 | cgggatccCCAGACTTGGAACACTCAGGAAT | ggaatccTGCATGCTCCATTTATTGAATTTG |
| siRNA- ADAMTS9-AS1-1# | AAUGUUCUCUAUUUUUGCCUU |  |
| siRNA- ADAMTS9-AS1-2# | AUGAGAAAGAGCUUAUGCCUU |  |
| siRNA- ADAMTS9-AS1-3# | UUCUCUUAUGAGAAAGAGCUU |  |
| siRNA-Gpx4-1# | AAUGAGAAACUUGGUAAAGUU |  |
| siRNA-Gpx4-2# | UGUACAUGUCAAACUUGACGU |  |
| miR-6516-5p mimic | UUUGCAGUAACAGGUGUGGACA |  |
| qPCR for ADAMS9-AS1 | AGATGTGAGGATAGCAAGGGGC | GGCAGAGGAATGGCAAGAAGT |
| qPCR for cox-2 | CTTTGGAGGCGAAGTGGGTT | GCTGTTTTGGTAGGCTGTGGAT |
| qPCR for Acsl4 | CATTATATTGCTGCCTGTCCACTT | GGGCTTAGCTTTTATTCTCTTTGC |
| qPCR for Nox1 | CACGAGTGGGATGACCATAAGG | CTGGGAGCGATAAAAGCGAAG |
| qPCR for Gpx4 | GGAACTTTACCAAGTTTCTCATTGAT | CACGGCAGGTCCTTCTCTATCA |
| qPCR for Fth1 | AGTTGTATGCCTCCTACGTCTATCTG | AGTCATCACGGTCTGGTTTCTTTA |
|  |  |  |
