# Supplementary material for: Long noncoding RNA ADAMTS9-AS1 represses ferroptosis of endometrial stromal cells by regulating the miR-6516-5p/GPX4 axis in endometriosis
Source: Sci Rep. 2022 Feb 16;12:2618. doi: 10.1038/s41598-022-04963-z (PMC8850595; doi:10.1038/s41598-022-04963-z)
Supplement: Supplementary file 4 — Supplementary Information 4. [file 41598_2022_4963_MOESM4_ESM.docx]

**Supporting Table S2.** The prediction of miRNAs interacted with ADAMTS9-AS1 using miRDB (http://mirdb.org/mirdb/index.html).

| **Target Detail** | **Target Rank** | **Target Score** | **miRNA Name** | **Gene Symbol** |
| --- | --- | --- | --- | --- |
|  | 1 | 94 | [hsa-miR-6516-5p](http://mirdb.org/cgi-bin/mature_mir.cgi?name=hsa-miR-6516-5p) | submission |
|  | 2 | 92 | [hsa-miR-3935](http://mirdb.org/cgi-bin/mature_mir.cgi?name=hsa-miR-3935) | submission |
|  | 3 | 88 | [hsa-miR-651-3p](http://mirdb.org/cgi-bin/mature_mir.cgi?name=hsa-miR-651-3p) | submission |
|  | 4 | 88 | [hsa-miR-1204](http://mirdb.org/cgi-bin/mature_mir.cgi?name=hsa-miR-1204) | submission |
|  | 5 | 88 | [hsa-miR-2467-3p](http://mirdb.org/cgi-bin/mature_mir.cgi?name=hsa-miR-2467-3p) | submission |
|  | 6 | 86 | [hsa-miR-130b-5p](http://mirdb.org/cgi-bin/mature_mir.cgi?name=hsa-miR-130b-5p) | submission |
|  | 7 | 85 | [hsa-miR-31-5p](http://mirdb.org/cgi-bin/mature_mir.cgi?name=hsa-miR-31-5p) | submission |
|  | 8 | 83 | [hsa-miR-3160-3p](http://mirdb.org/cgi-bin/mature_mir.cgi?name=hsa-miR-3160-3p) | submission |
|  | 9 | 83 | [hsa-miR-488-3p](http://mirdb.org/cgi-bin/mature_mir.cgi?name=hsa-miR-488-3p) | submission |
|  | 10 | 81 | [hsa-miR-4297](http://mirdb.org/cgi-bin/mature_mir.cgi?name=hsa-miR-4297) | submission |
|  | 11 | 81 | [hsa-miR-3163](http://mirdb.org/cgi-bin/mature_mir.cgi?name=hsa-miR-3163) | submission |
|  | 12 | 80 | [hsa-miR-6514-5p](http://mirdb.org/cgi-bin/mature_mir.cgi?name=hsa-miR-6514-5p) | submission |
|  | 13 | 80 | [hsa-miR-3674](http://mirdb.org/cgi-bin/mature_mir.cgi?name=hsa-miR-3674) | submission |
|  | 14 | 79 | [hsa-miR-4276](http://mirdb.org/cgi-bin/mature_mir.cgi?name=hsa-miR-4276) | submission |
|  | 15 | 76 | [hsa-miR-6809-3p](http://mirdb.org/cgi-bin/mature_mir.cgi?name=hsa-miR-6809-3p) | submission |
|  | 16 | 76 | [hsa-miR-653-3p](http://mirdb.org/cgi-bin/mature_mir.cgi?name=hsa-miR-653-3p) | submission |
|  | 17 | 76 | [hsa-miR-124-5p](http://mirdb.org/cgi-bin/mature_mir.cgi?name=hsa-miR-124-5p) | submission |
|  | 18 | 76 | [hsa-miR-6817-3p](http://mirdb.org/cgi-bin/mature_mir.cgi?name=hsa-miR-6817-3p) | submission |
|  | 19 | 75 | [hsa-miR-6799-5p](http://mirdb.org/cgi-bin/mature_mir.cgi?name=hsa-miR-6799-5p) | submission |
|  | 20 | 74 | [hsa-miR-6883-3p](http://mirdb.org/cgi-bin/mature_mir.cgi?name=hsa-miR-6883-3p) | submission |
|  | 21 | 74 | [hsa-miR-539-5p](http://mirdb.org/cgi-bin/mature_mir.cgi?name=hsa-miR-539-5p) | submission |
|  | 22 | 73 | [hsa-miR-2117](http://mirdb.org/cgi-bin/mature_mir.cgi?name=hsa-miR-2117) | submission |
|  | 23 | 72 | [hsa-miR-3926](http://mirdb.org/cgi-bin/mature_mir.cgi?name=hsa-miR-3926) | submission |
|  | 24 | 72 | [hsa-miR-490-5p](http://mirdb.org/cgi-bin/mature_mir.cgi?name=hsa-miR-490-5p) | submission |
|  | 25 | 70 | [hsa-miR-1200](http://mirdb.org/cgi-bin/mature_mir.cgi?name=hsa-miR-1200) | submission |
|  | 26 | 70 | [hsa-miR-664a-3p](http://mirdb.org/cgi-bin/mature_mir.cgi?name=hsa-miR-664a-3p) | submission |
|  | 27 | 70 | [hsa-miR-138-1-3p](http://mirdb.org/cgi-bin/mature_mir.cgi?name=hsa-miR-138-1-3p) | submission |
|  | 28 | 69 | [hsa-miR-194-3p](http://mirdb.org/cgi-bin/mature_mir.cgi?name=hsa-miR-194-3p) | submission |
|  | 29 | 69 | [hsa-miR-551b-5p](http://mirdb.org/cgi-bin/mature_mir.cgi?name=hsa-miR-551b-5p) | submission |
|  | 30 | 67 | [hsa-miR-154-5p](http://mirdb.org/cgi-bin/mature_mir.cgi?name=hsa-miR-154-5p) | submission |
|  | 31 | 67 | [hsa-miR-642b-5p](http://mirdb.org/cgi-bin/mature_mir.cgi?name=hsa-miR-642b-5p) | submission |
|  | 32 | 67 | [hsa-miR-4690-5p](http://mirdb.org/cgi-bin/mature_mir.cgi?name=hsa-miR-4690-5p) | submission |
|  | 33 | 66 | [hsa-miR-4790-3p](http://mirdb.org/cgi-bin/mature_mir.cgi?name=hsa-miR-4790-3p) | submission |
|  | 34 | 66 | [hsa-miR-449b-3p](http://mirdb.org/cgi-bin/mature_mir.cgi?name=hsa-miR-449b-3p) | submission |
|  | 35 | 66 | [hsa-miR-4503](http://mirdb.org/cgi-bin/mature_mir.cgi?name=hsa-miR-4503) | submission |
|  | 36 | 66 | [hsa-miR-552-5p](http://mirdb.org/cgi-bin/mature_mir.cgi?name=hsa-miR-552-5p) | submission |
|  | 37 | 65 | [hsa-miR-5581-5p](http://mirdb.org/cgi-bin/mature_mir.cgi?name=hsa-miR-5581-5p) | submission |
|  | 38 | 65 | [hsa-miR-4755-3p](http://mirdb.org/cgi-bin/mature_mir.cgi?name=hsa-miR-4755-3p) | submission |
|  | 39 | 64 | [hsa-miR-5580-5p](http://mirdb.org/cgi-bin/mature_mir.cgi?name=hsa-miR-5580-5p) | submission |
|  | 40 | 64 | [hsa-miR-95-5p](http://mirdb.org/cgi-bin/mature_mir.cgi?name=hsa-miR-95-5p) | submission |
|  | 41 | 64 | [hsa-miR-519e-5p](http://mirdb.org/cgi-bin/mature_mir.cgi?name=hsa-miR-519e-5p) | submission |
|  | 42 | 64 | [hsa-miR-515-5p](http://mirdb.org/cgi-bin/mature_mir.cgi?name=hsa-miR-515-5p) | submission |
|  | 43 | 64 | [hsa-miR-30e-3p](http://mirdb.org/cgi-bin/mature_mir.cgi?name=hsa-miR-30e-3p) | submission |
|  | 44 | 64 | [hsa-miR-30d-3p](http://mirdb.org/cgi-bin/mature_mir.cgi?name=hsa-miR-30d-3p) | submission |
|  | 45 | 64 | [hsa-miR-30a-3p](http://mirdb.org/cgi-bin/mature_mir.cgi?name=hsa-miR-30a-3p) | submission |
|  | 46 | 64 | [hsa-miR-6816-3p](http://mirdb.org/cgi-bin/mature_mir.cgi?name=hsa-miR-6816-3p) | submission |
|  | 47 | 63 | [hsa-miR-548c-3p](http://mirdb.org/cgi-bin/mature_mir.cgi?name=hsa-miR-548c-3p) | submission |
|  | 48 | 63 | [hsa-miR-6739-3p](http://mirdb.org/cgi-bin/mature_mir.cgi?name=hsa-miR-6739-3p) | submission |
|  | 49 | 62 | [hsa-miR-486-3p](http://mirdb.org/cgi-bin/mature_mir.cgi?name=hsa-miR-486-3p) | submission |
|  | 50 | 62 | [hsa-miR-206](http://mirdb.org/cgi-bin/mature_mir.cgi?name=hsa-miR-206) | submission |
|  | 51 | 62 | [hsa-miR-1-3p](http://mirdb.org/cgi-bin/mature_mir.cgi?name=hsa-miR-1-3p) | submission |
|  | 52 | 61 | [hsa-miR-371a-5p](http://mirdb.org/cgi-bin/mature_mir.cgi?name=hsa-miR-371a-5p) | submission |
|  | 53 | 61 | [hsa-miR-642a-5p](http://mirdb.org/cgi-bin/mature_mir.cgi?name=hsa-miR-642a-5p) | submission |
|  | 54 | 61 | [hsa-miR-4766-3p](http://mirdb.org/cgi-bin/mature_mir.cgi?name=hsa-miR-4766-3p) | submission |
|  | 55 | 60 | [hsa-miR-7113-5p](http://mirdb.org/cgi-bin/mature_mir.cgi?name=hsa-miR-7113-5p) | submission |
|  | 56 | 60 | [hsa-miR-548s](http://mirdb.org/cgi-bin/mature_mir.cgi?name=hsa-miR-548s) | submission |
|  | 57 | 60 | [hsa-miR-6833-5p](http://mirdb.org/cgi-bin/mature_mir.cgi?name=hsa-miR-6833-5p) | submission |
|  | 58 | 59 | [hsa-miR-1252-5p](http://mirdb.org/cgi-bin/mature_mir.cgi?name=hsa-miR-1252-5p) | submission |
|  | 59 | 59 | [hsa-miR-7-2-3p](http://mirdb.org/cgi-bin/mature_mir.cgi?name=hsa-miR-7-2-3p) | submission |
|  | 60 | 59 | [hsa-miR-7-1-3p](http://mirdb.org/cgi-bin/mature_mir.cgi?name=hsa-miR-7-1-3p) | submission |
|  | 61 | 58 | [hsa-miR-10527-5p](http://mirdb.org/cgi-bin/mature_mir.cgi?name=hsa-miR-10527-5p) | submission |
|  | 62 | 58 | [hsa-miR-6813-3p](http://mirdb.org/cgi-bin/mature_mir.cgi?name=hsa-miR-6813-3p) | submission |
|  | 63 | 58 | [hsa-miR-520f-5p](http://mirdb.org/cgi-bin/mature_mir.cgi?name=hsa-miR-520f-5p) | submission |
|  | 64 | 58 | [hsa-miR-5681a](http://mirdb.org/cgi-bin/mature_mir.cgi?name=hsa-miR-5681a) | submission |
|  | 65 | 57 | [hsa-miR-6730-5p](http://mirdb.org/cgi-bin/mature_mir.cgi?name=hsa-miR-6730-5p) | submission |
|  | 66 | 57 | [hsa-miR-138-2-3p](http://mirdb.org/cgi-bin/mature_mir.cgi?name=hsa-miR-138-2-3p) | submission |
|  | 67 | 56 | [hsa-miR-6499-3p](http://mirdb.org/cgi-bin/mature_mir.cgi?name=hsa-miR-6499-3p) | submission |
|  | 68 | 56 | [hsa-miR-4789-3p](http://mirdb.org/cgi-bin/mature_mir.cgi?name=hsa-miR-4789-3p) | submission |
|  | 69 | 56 | [hsa-miR-575](http://mirdb.org/cgi-bin/mature_mir.cgi?name=hsa-miR-575) | submission |
|  | 70 | 56 | [hsa-miR-5197-5p](http://mirdb.org/cgi-bin/mature_mir.cgi?name=hsa-miR-5197-5p) | submission |
|  | 71 | 55 | [hsa-miR-6841-5p](http://mirdb.org/cgi-bin/mature_mir.cgi?name=hsa-miR-6841-5p) | submission |
|  | 72 | 55 | [hsa-miR-519e-3p](http://mirdb.org/cgi-bin/mature_mir.cgi?name=hsa-miR-519e-3p) | submission |
|  | 73 | 55 | [hsa-miR-515-3p](http://mirdb.org/cgi-bin/mature_mir.cgi?name=hsa-miR-515-3p) | submission |
|  | 74 | 55 | [hsa-miR-33b-3p](http://mirdb.org/cgi-bin/mature_mir.cgi?name=hsa-miR-33b-3p) | submission |
|  | 75 | 55 | [hsa-miR-6763-5p](http://mirdb.org/cgi-bin/mature_mir.cgi?name=hsa-miR-6763-5p) | submission |
|  | 76 | 55 | [hsa-miR-3150a-3p](http://mirdb.org/cgi-bin/mature_mir.cgi?name=hsa-miR-3150a-3p) | submission |
|  | 77 | 54 | [hsa-miR-10523-5p](http://mirdb.org/cgi-bin/mature_mir.cgi?name=hsa-miR-10523-5p) | submission |
|  | 78 | 54 | [hsa-miR-613](http://mirdb.org/cgi-bin/mature_mir.cgi?name=hsa-miR-613) | submission |
|  | 79 | 54 | [hsa-miR-4742-3p](http://mirdb.org/cgi-bin/mature_mir.cgi?name=hsa-miR-4742-3p) | submission |
|  | 80 | 54 | [hsa-miR-589-3p](http://mirdb.org/cgi-bin/mature_mir.cgi?name=hsa-miR-589-3p) | submission |
|  | 81 | 54 | [hsa-miR-4452](http://mirdb.org/cgi-bin/mature_mir.cgi?name=hsa-miR-4452) | submission |
|  | 82 | 54 | [hsa-miR-4266](http://mirdb.org/cgi-bin/mature_mir.cgi?name=hsa-miR-4266) | submission |
|  | 83 | 53 | [hsa-miR-4753-3p](http://mirdb.org/cgi-bin/mature_mir.cgi?name=hsa-miR-4753-3p) | submission |
|  | 84 | 53 | [hsa-miR-6809-5p](http://mirdb.org/cgi-bin/mature_mir.cgi?name=hsa-miR-6809-5p) | submission |
|  | 85 | 53 | [hsa-miR-6870-5p](http://mirdb.org/cgi-bin/mature_mir.cgi?name=hsa-miR-6870-5p) | submission |
|  | 86 | 53 | [hsa-miR-3120-5p](http://mirdb.org/cgi-bin/mature_mir.cgi?name=hsa-miR-3120-5p) | submission |
|  | 87 | 53 | [hsa-miR-4766-5p](http://mirdb.org/cgi-bin/mature_mir.cgi?name=hsa-miR-4766-5p) | submission |
|  | 88 | 53 | [hsa-miR-3613-3p](http://mirdb.org/cgi-bin/mature_mir.cgi?name=hsa-miR-3613-3p) | submission |
|  | 89 | 52 | [hsa-miR-5688](http://mirdb.org/cgi-bin/mature_mir.cgi?name=hsa-miR-5688) | submission |
|  | 90 | 52 | [hsa-miR-8075](http://mirdb.org/cgi-bin/mature_mir.cgi?name=hsa-miR-8075) | submission |
|  | 91 | 52 | [hsa-miR-1299](http://mirdb.org/cgi-bin/mature_mir.cgi?name=hsa-miR-1299) | submission |
|  | 92 | 52 | [hsa-miR-3912-5p](http://mirdb.org/cgi-bin/mature_mir.cgi?name=hsa-miR-3912-5p) | submission |
|  | 93 | 52 | [hsa-miR-8053](http://mirdb.org/cgi-bin/mature_mir.cgi?name=hsa-miR-8053) | submission |
|  | 94 | 52 | [hsa-miR-6740-3p](http://mirdb.org/cgi-bin/mature_mir.cgi?name=hsa-miR-6740-3p) | submission |
|  | 95 | 52 | [hsa-miR-7152-5p](http://mirdb.org/cgi-bin/mature_mir.cgi?name=hsa-miR-7152-5p) | submission |
|  | 96 | 51 | [hsa-miR-6079](http://mirdb.org/cgi-bin/mature_mir.cgi?name=hsa-miR-6079) | submission |
|  | 97 | 51 | [hsa-miR-580-5p](http://mirdb.org/cgi-bin/mature_mir.cgi?name=hsa-miR-580-5p) | submission |
|  | 98 | 51 | [hsa-miR-6823-3p](http://mirdb.org/cgi-bin/mature_mir.cgi?name=hsa-miR-6823-3p) | submission |
|  | 99 | 51 | [hsa-miR-578](http://mirdb.org/cgi-bin/mature_mir.cgi?name=hsa-miR-578) | submission |
|  | 100 | 51 | [hsa-miR-1266-3p](http://mirdb.org/cgi-bin/mature_mir.cgi?name=hsa-miR-1266-3p) | submission |
|  | 101 | 50 | [hsa-miR-3978](http://mirdb.org/cgi-bin/mature_mir.cgi?name=hsa-miR-3978) | submission |
|  | 102 | 50 | [hsa-miR-4531](http://mirdb.org/cgi-bin/mature_mir.cgi?name=hsa-miR-4531) | submission |
|  | 103 | 50 | [hsa-miR-153-5p](http://mirdb.org/cgi-bin/mature_mir.cgi?name=hsa-miR-153-5p) | submission |
|  | 104 | 50 | [hsa-miR-152-5p](http://mirdb.org/cgi-bin/mature_mir.cgi?name=hsa-miR-152-5p) | submission |
